# Supplementary material for: Activated Carbon from Palm Date Seeds for CO2 Capture
Source: Int J Environ Res Public Health. 2021 Nov 19;18(22):12142. doi: 10.3390/ijerph182212142 (PMC8624853; doi:10.3390/ijerph182212142)
Supplement: Supplementary file 1 [file ijerph-18-12142-s001.zip › ijerph-1437946-supplementary.pdf]

Activated Carbon from Palm Date Seeds for CO_2 capture

Amira Alazmi[1,*], Sabina A. Nicolae[2], Pierpaolo Modugno[2], Bashir E. Hasanov[3], Maria M. Titirici[4] and Pedro M. F. J. Costa[3]

1 Department of Chemistry, University Colleges at Nairiyah, University of Hafr Albatin (UHB), Nairiyah 31981, Saudi Arabia
2 School of Engineering and Materials Science, Queen Mary University of London, London E1 4NS, United Kingdom
3 King Abdullah University of Science and Technology (KAUST), Physical Science and Engineering Division, Thuwal 23955-6900, Saudi Arabia
4 Department of Chemical Engineering, Imperial College London, London SW7 2AZ, United Kingdom
** Correspondence: amira.alazmi@uhb.edu.sa*

Table S1. Additional Raman spectral data, along with the I_D/I_G (intensity ratio of D and G peaks), of the activated HTC-PDS materials.

Sample	D peak position	G peak position	2D peak position	I_D/I_G
HTC-PDS-CO_2	1334	1587	2723	1.1
HTC-PDS-KOH_1	1329	1564	2656	0.8
HTC-PDS-KOH_2	1334	1583	2660	1.1
HTC-PDS-H_3PO_4_1	1338	1585	2729	1.0
HTC-PDS-H_3PO_4_2	1338	1580	2729	1.1

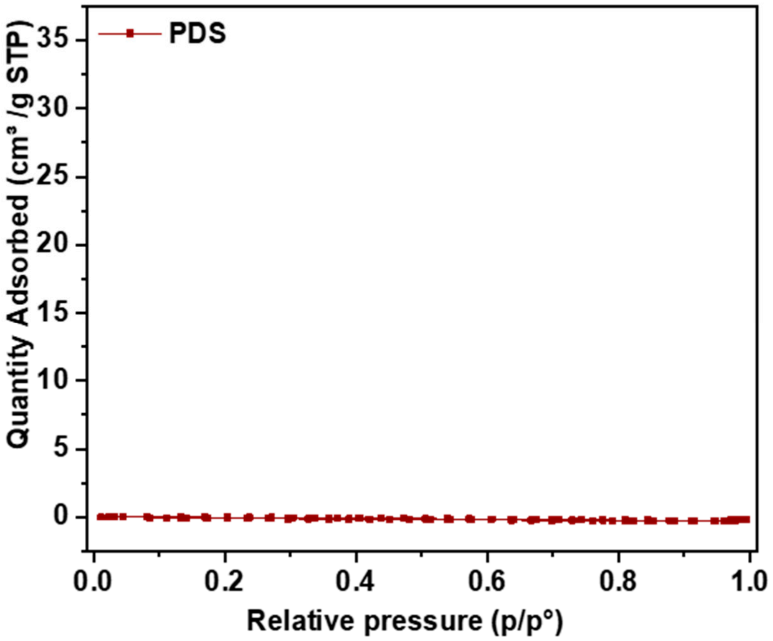

Figure S1. N_2 sorption isotherms of the PDS powder (at -196 °C).

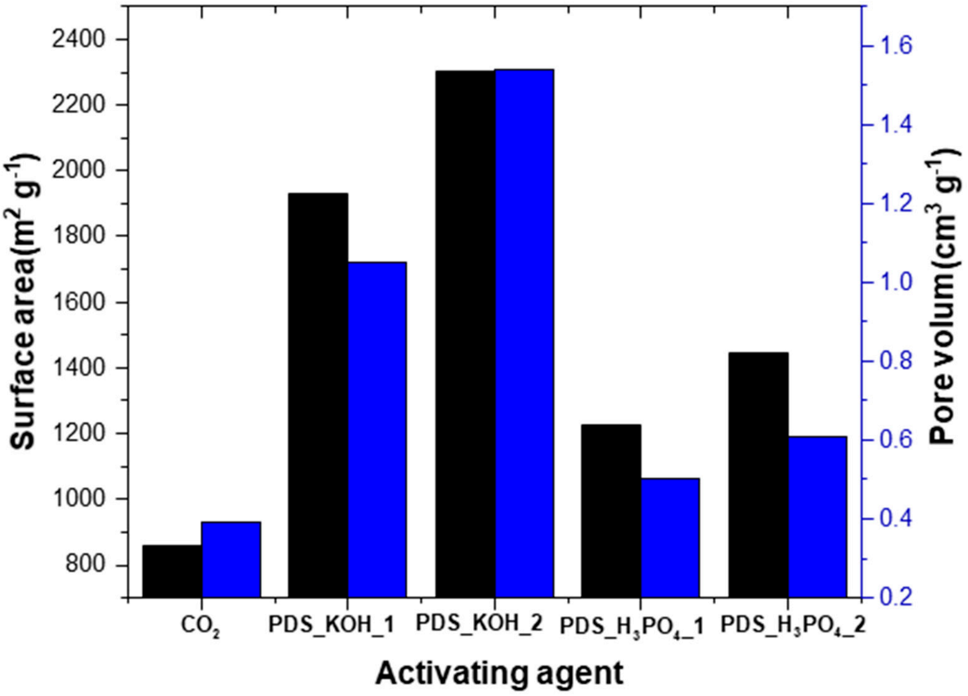

Figure S2. Effect of the activating agents on the pore structure of the HTC-PDS materials.

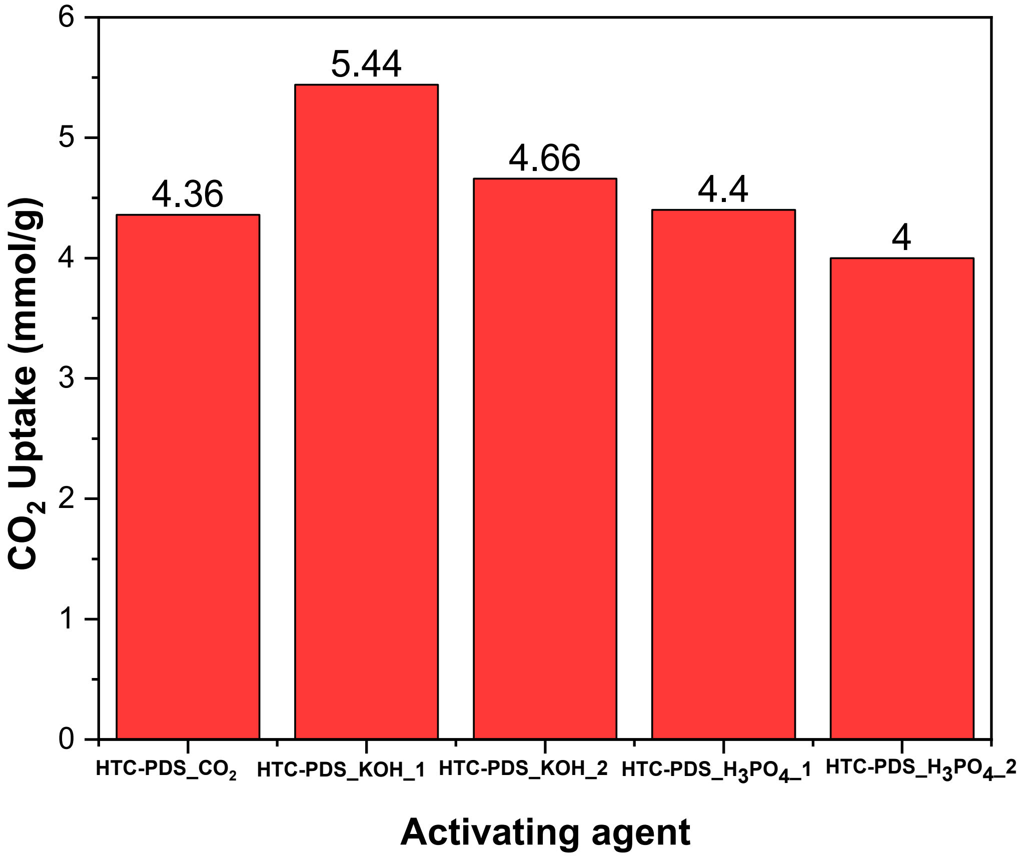

Figure S3. Comparison of maximum CO_2 uptake (at 1 bar) for the different activated samples in **Figure 4**.
